# Personal Freedom and Social Responsibility in Slowing the Spread of COVID-19: A Rapid Qualitative Study

**DOI:** 10.1177/10901981211033241

**Published:** 2022-02

**Authors:** Evelyn Vázquez, Julie Chobdee, Niloufar Nasrollahzadeh, Ann Cheney

**Affiliations:** 1 University of California, Riverside, CA, USA; 2 University of Southern California, Los Angeles, CA, USA

**Keywords:** college, college health, community health promotion, health education, socioecological model

## Abstract

This rapid qualitative exploratory study focused on perceptions of adopting risk-reduction measures, such as face masks, on campuses within institutions of higher education in the United States. It was intended to identify safety measures to reduce virus spread and develop community-informed public health messaging to promote COVID-19 risk-reduction strategies within campus communities. This study was approved by the institutional review board where the study took place. A total of 113 stakeholders, including students, staff, and faculty attended one of nine focus groups. We use the socioecological model to illustrate the use of COVID-19 public health measures in private and public spaces and how macro-level processes, specifically sociocultural values of personal freedom and social responsibility shape the meaning and interpretation of COVID-19 public health measures. A rapid qualitative data analysis was conducted. This analysis was characterized by three steps: (1) transcription of the interviews, (2) completion of a summary template per focus group analysis (data reduction strategy), and (3) matrix analyses involving a cross-case analysis of the nine focus groups conducted. Based on study findings, we offer community-centered recommendations for safe and healthy reopening of large public research institutions. This article contributes to the foundation of scientific literature that qualitatively describes evidence-based strategies for safe reopening of places of education and employment in the COVID-19 pandemic.

The COVID-19 pandemic has contributed to widespread social and economic change across the globe and significantly increased risk for illnesses and death. In the United States, the pandemic rapidly changed everyday practices and policies within learning and working spaces, closing down businesses, public services, and educational institutions. As organizations now prepare for in-person learning and work, made possible by the emergency approval of COVID-19 vaccines, organizational leadership must consider the health concerns and needs of its community. This is especially the case for institutions of higher education (IHEs).

As anchor institutions, IHEs serve local communities. Approximately 19.7 million students are enrolled at U.S. colleges that employ over 3 million people (National Center for Education Statistics, 2021). IHEs also offer access to cultural and social events, recreational spaces, and public learning (Scholl & Gulwadi, 2015). As a result of the COVID-19 outbreak, most U.S. campuses fully or partially closed, moving instruction, research, and businesses practices to the online environment. U.S. Centers for Disease Control and Prevention (CDC, 2020) guidelines describe how IHEs can protect their communities and slow the spread of COVID-19, including use of face coverings (both on and off campus), hand hygiene, and social distancing measures. However, to date, there is limited information grounded in campus community members’ concerns and needs about the adoption of COVID-19 risk-reduction measures and recommendations for safe campus reopening.

Navigating the COVID-19 pandemic and its aftermath will be challenging for organizations, including IHEs. Throughout the pandemic, organizations rapidly employed new ways of working and business operations while minimizing risk for COVID-19. The field of workplace wellness offers some insight into safe reopening plans (Henderson, 2021). Leadership must consider the occupational health of their employees in the transition from remote to in-person work. They must consider back-to-work safety protocols and policies that promote health and safety, maximize social distancing (e.g., spacing of workstations), and visual social distancing and sanitation cues. Furthermore, personal and workplace safety and hygiene protocols need to be followed. Such safety measures reduce exposure to COVID-19 and are modifiable risk factors that can help protect employees as well as reduce stress on returning to work in the pandemic (Evanoff et al., 2020; Tan et al., 2020).

To date, much of what we know about returning to in-person interaction (e.g., work) in the context of COVID-19 is based on the perceptions and experiences of health care workers and other essential workers. Given the need for exploratory data on this topic among those embedded within IHEs, we conducted a rapid qualitative research study in a public research university in the western United States. The purpose of this study was to identify safety measures to reduce virus spread and describe community-informed public health messaging to promote COVID-19 risk-reduction strategies within campus communities. The following questions guided the research: What are the perceptions of student, staff, and faculty about adopting risk-reduction measures, such as face coverings, on campuses within IHEs? What are their recommendations for safe reopening and return to campus?

## Theoretical Framework

Socioecological models (SEMs) are used to explore how changes in the social environment promote changes in individual behaviors (McLeroy et al., 1988). These models permit identification of macro-level (e.g., social, cultural, economic, and political) causes of poor and unhealthy behaviors, and the reciprocal relationship between individuals and their environment. Scholars have developed ecological models for health promotion focused on complementary and intersecting factors, including the microsystem of intrapersonal characteristics (e.g., self-concepts) and interpersonal processes (e.g., working groups), the meso- or exosystem including community factors (e.g., resources and relationships among organizations), and macrosystem involving laws and public policy (Dahlberg & Krug, 2002; Roura, 2020).

The SEM explains the interplay of interpersonal relationships (micro) with a sense of community and organizational characteristics (meso) and institutional policy (macro), thus informing the steps needed to promote environmental change and health promotion in IHEs (Golden & Earp, 2012). The microsystem plays a significant role in health behaviors by providing social resources, such as emotional support and access to new social roles and identities, which can mediate life stress (Wills, 1985). Organizations allow individuals the opportunity to build social and emotional support for collective change. Organizational characteristics (e.g., incentives, regulations), support behavioral changes. The sense of community within organizations can mediate macro-level processes and establish or strengthen ties among individuals.

In the context of the current pandemic, the intersection of micro-, meso-, and macro-level processes influence the norms and values of communities as well as individual-level attitudes, knowledge, and behaviors. This intersection also impacts policy-making processes, especially those intended to protect community health. We use this framework to highlight the intersecting factors of macro-level processes, specifically sociocultural factors, that shape ideas about COVID-19 public health measures and the adoption of safety measures in higher education settings.

## Method

### The Study Site

The analyses presented in this article are based on a 6-month study of campus perceptions of safety measures to reduce risk for COVID-19 in IHEs. We conducted research with students, staff, and faculty attending or working at a 4-year public research IHE located in southern California. This university consists of approximately 20,500 undergraduate and 3,350 graduate and professional students, 8,800 staff, and 1,950 faculty. The investigative team were members of our campus’ public health collaborative focused on creating cultures of health (Cheney et al., 2020).

The public health measures taken by the campus were shaped by the shelter-in-place and stay-at-home orders from the state of California, including campus closure with most interactions held remotely (e.g., online classes and home office telework). In summer 2020, at the time of this study, the state of California had over 500,000 COVID-19 cases and led the nation in total cases by state/territory (Allen et al., 2021). According to the California Department of Public Health COVID-19 updates, at the time of this research, the county where the university is located had the second highest confirmed COVID-19 cases and deaths in the state (California Department of Public Health, 2020).

### Rapid Qualitative Data Collection and Analysis

Qualitative methods are ideal for exploring shared ideas and strategies to promote public health responses (Vindrola-Padros et al., 2020). We employed rapid data collection and analytic techniques involving focus groups and template and matrix analysis.

#### Recruitment and Inclusion Criteria

We followed two sampling techniques: purposeful and convenience. Participants were eligible if they were (1) 18 years of age or older and (2) current member of the campus community (student, staff, or faculty). We used purposive sample to recruit participants through emails sent out via campus listservs, posts on social media sites, and existing campus partnerships. Over 350 members of the campus community replied to the recruitment call. We then used convenience sampling, reaching out to the first individuals on the list and inviting them to attend. We invited approximately 150 campus members (60 students, 45 staff, 45 faculty) to attend one of the nine focus groups. We sought 10 participants per focus group. To account for attrition, we oversampled students, a decision based on previous research with students involving low recruitment rates. However, for this study, nearly all invited students attended a focus group.

#### Consent and Incentives

Prior to the start of research, this study was approved by the Institutional Review Board. All survey participants provided electronic consent; focus group participants provided verbal consent. Focus group participants were given the option to receive a $10 gift card for their participation in the study or donate it to campus COVID-19 efforts.

#### Focus Groups

In September 2020, we conducted nine focus groups, three with students (*n* = 42), three with staff (*n* = 41), and three with faculty (*n* = 30), totaling 113 participants. Focus groups allow participants to build on each other’s ideas, and in this study, they provided collective (rather than individual) information about COVID-19 in university settings (Krueger & Casey, 2000). Because 80% of themes can be identified within two to three focus groups, we conducted three focus groups per stakeholder group (Guest et al., 2017).

Trained facilitators used a semistructured interview guide exploring: (1) thoughts on the virus, (2) perceptions on safety measures, and (3) recommendations for safely returning to campus. A facilitator and note taker attended each focus group. Focus groups were conducted via Zoom video conferencing and lasted approximately 60 to 90 minutes.

#### Template and Matrix Analysis

Rapid qualitative data analysis allowed us to identify patterns across the nine focus groups (Beebe, 2014; Creswell, 2013). This analysis was characterized by three steps: (1) transcription of the interviews, (2) completion of a summary template per focus group (data reduction strategy), and (3) matrix analyses involving a cross-case analysis of the focus groups.

We used a professional service to transcribe the audio recordings. Team members listened to the recordings and corrected transcription errors. They read the transcripts line by line and inserted data, including exemplar quotes from the group interviews, in the summary templates. Next, we created a matrix, which included focus groups (as rows) and interview topics (as columns). Team members inserted data from each summary template into matrices, one per stakeholder group (students, staff, faculty) for comparative purposes. These matrices helped condense and manage the data facilitating theme identification across stakeholder groups (Averill, 2002).

Three credibility strategies—researcher’s reflexivity, peer examination, and triangulation—were used to increase trustworthiness of the data (Krefting, 1991; Patton, 2002). Notetaker templates captured reflexive notes and analytical reflections that responded to the goals of the investigation. For the peer review, team members reviewed the transcriptions and constructed the matrix contributing to the identification of themes and final analysis (Creswell, 2007). All members of the analytical team established a dialectical and iterative relationship in which they met constantly as well as exchanged dialogue about their reflections and insights on the meaning of the data. Finally, triangulation via data sources involved the use of a wide range of participant perspectives, including those of students, staff, and faculty (Shenton, 2004).

## Results

The presentation of our findings maps onto the SEM. We first discuss the intersection of macro-level factors with community and individual experiences of COVID-19, focusing on perceived risk in public and private spaces. We then discuss perceptions of COVID-19 safety measures, highlighting sociocultural processes of American societal values of personal freedom and social responsibility. Finally, we highlight campus community recommendations for safe reopening of large public research institutions.

### Individual Experiences of COVID-19

Most students, staff, and faculty who participated in our study worked or studied from home; only a few accessed on-campus office spaces or facilities. Their narratives focus on experiences in public and private spaces in their community and household, as well as with colleagues in campus settings. Participants discussed the mental health burden linked to the stress and uncertainty of the pandemic, challenges with adhering to public health measures, and the abrupt shift to working or studying from home, which for some also involved raising children and caring for others. As discussed below, participants’ perceived risk changed depending on the spaces they occupied.

#### Public Spaces

A theme that emerged across all focus groups was a sense of risk for contracting COVID-19 based on other people’s behaviors in public spaces. One of the main sources of discomfort was the lack of mask wearing and social distancing in public spaces, especially grocery stores (perceived as critical to access). While participants generally expressed concerns for risk in grocery stores, students were especially critical of patrons’ behaviors and stores’ management policies around the proper use of public health guidelines. Participants (see Table 1 for exemplar quotes) also expressed concern about social distancing in grocery stores: “It’s really hard to social distance in grocery stores, which is honestly kind of the only other place that I go outside of my house.”

**Table 1.** COVID-19 Experiences and Perceived Risk.

Theme	Perceived risk		Stakeholders’ lived experiences
COVID-19 Experiences and Perceived Risk	Public spaces	Grocery stores	All the grocery stores in California require you to wear a mask. So, once people enter the store, I see them wearing the mask; but once they’re inside the aisles, when they’re purchasing [products], I see people taking away their masks because there’s nobody to check [them]. So, I think that’s bad. I think there should be someone to check that they’re all wearing the mask, even inside the store.
		Social distancing	I do observe a lot of people who are really stressed out when they’re out in public now. I’ve seen public confrontation of people at Costco, for instance, about people wearing a mask and not wearing a mask. And I’ve seen physical altercations over masks. . . . a lot of people are more on edge. It’s a lot more of a stressful situation of also taking your kids out—you know, parents yelling at their kids not to touch the floor, “Don’t touch anything in the supermarket.” It’s definitely a heightened experience for everyone in public right now.
	Private spaces		I’ve had the most uncomfortable question: “Do you want me to put my mask on?” I’ve had people say that to me. I’m like: “Hell yes I do.” . . . I just find that [to be] a very startling question. “How are we still asking that right now?” Cause if the answer was no, we’d all be back together and doing things.
	Public health (mis)information	Masks or face coverings limit ability to breathe	A lot of people don’t wear masks because they think like: “It’s annoying to wear.” or, “It’s hard to breathe in it.” It’s frustrating when they say that, because there’s people in hospitals right now that are intubated, and they need to be on a breathing machine. And, you’re complaining that it’s hard to breathe with the mask on? That’s frustrating that people are being selfish in that way. The purpose of the mask is to prevent possibly you from spreading it to others.

#### Private Spaces

Another source of perceived risk occurred in private spaces, particularly, with close friends, family, housemates, and teammates. Participants discussed being in these spaces with others who did not wear masks or questioned their use. Participants shared that younger generations perceive themselves as less vulnerable to the disease and its potential severity.

### Perceptions of COVID-19 Safety Measures

Participants were aware of COVID-19 safety measures to reduce virus spread, and most followed public health guidelines. However, they recognized the challenges with widespread use of COVID-19 safety measures. They commented that misinformation, spread through social media outlets and news channels, impedes use of public health measures intended to slow virus spread. Consumption of misinformation was more likely to occur among young people via social media and older adults via traditional channels (television news). Misinformation can lead to improper or no use of safety measures. Participants discussed how the belief that masks or face coverings limit ability to breathe, shaping attitudes about public health recommendations and leading some to not wear masks or wear them incorrectly. Additionally, participants discussed how the value of personal freedom outweighed social consciousness and the shared responsibility to protect self and others (see Table 2 for exemplar quotes).

**Table 2.** Values Shaping Use of Public Health Safety Measures.

Theme	Community-centered strategies		Stakeholders’ lived experiences
Values Shaping Use of Public Health Safety Measures	Personal freedom	Wearing a mask as a personal choice	There’s this emerging perspective that wearing a mask is a personal choice . . . for example, a distant uncle of mine in the Midwest just passed away. I was reading his obituary and for the Memorial service, masks are optional as it is a personal choice. [It] is how they framed it there. This is super puzzling to me. It’s super opposed to this idea of social responsibility—that, it’s not a personal choice. But it’s a choice that you make for your friends, your neighbors, for everybody else. I view this as a big barrier to why people are not wearing masks because they are framing it as something that I can choose to do or not do for myself.
		Freedom	I see that she’s [friend] all about politics: She wants to be free. She doesn’t think she needs to wear a mask. I disagree. I don’t think it’s at all about freedom. I think it’s about being considerate to everyone in every situation. I agree with all of you; it really needs to be about consideration for others, not your considerations.
	Social responsibility	Social responsibility	I’ve seen on the news, people protesting with posters saying, “My job is not to protect you.” . . . “My rights to not wear a mask or to carry a gun, or etc. are more important.”. . . I think that’s a huge difference in our culture versus Europe, or let’s say Singapore,—about personal freedom. The idea that somebody feels it’s not their civil responsibility to protect their country people, countrymen. . . . I have a very hard time personally understanding that.
		Frustration	I want to go and meet my friends and I would love to go and have a bunch of fun outside right now, but I feel like everyone has their own responsibility towards the general public health. And it’s just frustrating to see people not social distancing, [to] not wear masks and be in close proximity to each other, when other people are going out of their way to quarantine and stay home for the general well-being of people.
		Social and emotional needs versus the risk of COVID-19	It’s been a balance of the emotional, mental health needs, sort of what [another participant] said in the chat, versus the risks of COVID most of the time. Like, if I meet up with someone it’s outdoors, socially distant, and I’m personally comfortable. If we’re socially distanced and outdoors, we don’t need to wear masks. but if we get closer, move around at all, then we put the masks on.
		Stress and isolation	We hear a lot about how stressed out our students are and I’m not hearing enough about how we’re working together as staff to deal with our own stresses. . . . I feel very isolated, like [another participant] said, my husband and my two grown children who live at home, both work in the service industry. So, they go off to work and I’m here by myself most of the time, which is hard for me.
		Living with someone at high risk for contracting COVID-19	For me, it’s been a weird situation because I live with my mom and she is at high risk, due to her respiratory problems. And so, it’s been very stressful at the beginning. I kind of was like: “I don’t want to see anybody. I’m trying to avoid everything just to be safe.” And then now as we slowly have reopened, I still feel stressed out about that. Like, I know that I’m doing the best to be safe, wear a mask, you know, have hand sanitizer, stuff like that. But I’m scared of that [of returning to campus].

#### Personal Freedom

The idea of personal freedom emerged in several focus groups. In this context, face coverings have been assigned symbolic meaning—they demonstrate people’s values and political viewpoints and stances. Participants emphasized the uniqueness of this value to American society, which shapes individual choices to wear face coverings: “I am free to choose whether I wear a mask reflects that American view on freedom.”

#### Social Responsibility

For many, the idea of mask wearing as a personal choice stood in contrast to mask wearing as a social responsibility—to “wear a mask to keep others around me safe and healthy.” The values shaping personal decisions to wear or not wear masks have consequences. For those choosing not to wear a mask, the decision contributes to virus spread and prompts those engaging in safety measures to identify additional safety strategies. For instance, participants pointed out that those who were socially conscious and chose to protect themselves and others had to change their routines, limit their time outside, and alter their behaviors, avoiding crowded places, friends, and loved ones.

Participants also discussed the stress of balancing their social and emotional needs versus the risk of COVID-19. Participants talked about “isolation bubble(s)” (when people socialize with a specific small group of other people, they knew were following COVID-19 safety recommendations) as an alternative to balance those needs. Participant stress linked to the pandemic also emerges when living with someone at high risk for contracting COVID-19.

### Recommendations for Safe Reopening: Campus’ Return to the “New Normal”

As detailed in Table 3, without successful implementation of safety measures, participants refused to return to campus. Participants indicated need for transparent regulations and enforcement of precautions for community members’ safety and comfort. They also encouraged partnerships with the county public health department for COVID-19 education and resources.

**Table 3.** Strategies to Improve Health Status During the COVID-19 Pandemic.

Theme	Community-centered strategies		Lived experience
Campus’ Return to the “New Normal”	Off-campus collaborations		[The university] is one of the pillars of the county who provides not only education, also health and well-being throughout the county. More collaboration between the [county] Health Department and [the university] is an absolute necessity. We have to have all of the forces together to provide high quality education along with the medical. You’re dealing with the well-being of human individuals and all of the residents in the county. So therefore, [the university] is not only responsible for the student faculty and the staff population, they’re also responsible for the county because we are a major educational institution who can provide and facilitate education for the county.
	Adherence to public health guidelines	Education on mask wearing	Well, it is also important to teach people that a lot of the time, the face mask does alter your oxygen, but it does not alter your breathing, unless you have a health condition. A lot of the time it’s mostly psychological. Like “claustrophobia” . . . it’s a real feeling for them [those who have claustrophobia]. So, it’s important for people to get used to it and to have a campaign showing people the right way of doing it [wearing masks], or how to deal with the anxiety of having the face mask.
		Hand sanitizer	I think the university would really love to bring people on campus, but it’s very concerning right now, there’s some locations on campus that don’t even have basic sanitation . . . How many times have we gone to restrooms and they don’t have a hand sanitizer? That’s basic sanitation. So, until the university starts pouring money into making sure that our facilities are actually equipped to hold the amount of people . . .
		Establishing proper disposal of personal protective equipment (PPE)	Let’s just say study areas and all that—maybe we work towards minimizing space, putting up more plexiglass shields in certain areas. . . . And then having more access to things like hand sanitizers or washing stations. . . . Maybe the new thing is going to be hand washing stations. . . . Oh, and trash cans, because . . . personal protective equipment, it’s becoming such an issue right now with trash. I think we’re gonna need more disposal and safe disposal areas, not open trash cans for like birds to fly in and pick out stuff.
		Six-feet markers	Have a lot of like those six foot markers, to have people be aware at all times. . . . I know some restaurants in Germany have started putting these pool noodles on their [customer’s] head, that looks ridiculous, but it’s a good marker . . . So if you put, I don’t know, pictures of basketball players or six feet people you’ll know, maybe that’s like a humorous way to let people know: “This is what a six foot radius looks like.” . . . People can imagine if you have an easy way for people to picture what six feet looks like.
		School spirit and community	The concern [for me] is, what happens once the students leave campus? So, how can we have them bring good practices to the socializing that they’re going to be doing? They’re going to be socializing. So, how can we make it like a school spirit kind of thing? “We’re in this together kind of idea” . . . How can we give the students permission to wear those masks? You know, to kind of counteract any kind of negative perception that they might be feeling, whether there’s one there or not like, “Oh, let’s make it cool to wear masks.” Let’s make it cool to wear masks, even when we’re just socializing with a few friends, which is also a place where the spread can happen.
	Flexible classrooms and office spaces	Hybrid classroom	So, going back, partially in person, partially hybrid or remote, that way you’re just limiting the number of students, you know, all large lecture classes, [such as] ethnic studies, intro to social history, those [courses] just should all be taught online from this point forward until we have a viable vaccine that works.
		Zoom fatigue	From what I’m hearing, other departments are not as consistent without the micromanaging, saying: “If you’re not here, I don’t believe you’re working. Granted, some people have to be there [on campus] because of the nature of their jobs, but having that trust of employees to be working [from home], which I think everybody is, if not working more now that we’re at home.

To ensure the safety of the campus community, participants suggested a number of structural interventions, including no-cost rapid COVID-19 testing, sanitation materials, cleaning supplies, increased indoor ventilation, and outdoor seating. They also suggested flexible work and class policies to allow students to meet their academic needs and staff and faculty to have flexible schedules to accommodate their employment and childcare or caretaking responsibilities during the pandemic and transitional phases.

#### Adherence to Public Health Guidelines

Participants suggested a top-down approach to promote public health safety measures and institutional policies implemented across the campus, as well as a ground-up approach to widespread adoption and adherence to public health measures.

Top-down approaches focused on policies and protocols developed by campus administration to enforce COVID-19 public health behaviors, given the outbreaks on college campuses across the nation. Administration needs to “set the tone for the entire year.” They emphasized being strict with mask wearing and consequences for those who do not follow guidelines. Participants discussed the need for mask etiquette, education on the effectiveness of masks, and instruction on masks, as well as the need for the campus to invest in sanitation. Others discussed the need for plexiglass shields in highly frequented campus areas, increased access to masks and sanitation stations across campus, and proper disposal of personal protective equipment (PPE). Participants also suggested signage to enforce social distancing and open spaces and outdoor meeting to ensure people stay 6 feet apart. They recommended that closed spaces (e.g., elevators) be limited to one person at a time and use of stairs, where possible, should be encouraged.

Ground-up approaches focused on how to increase adoption and proper use of face coverings among the campus community, (e.g., a “mask design competition,” developing information such as videos about how to properly wear a mask, and suggestions on how to fit a mask for comfort). Participants discussed creating a contest in which the student body or campus community could design their own masks as a community building activity: “Some sort of community building [. . .] embracing this, ‘I challenged together’ messaging would work well.”

Prior to opening, participants suggested that the university hold a “transitional [period]” to be “half in person and half [via] zoom.” This approach could increase confidence among campus community members by demonstrating that the university cares about individual and campus health and increase transparency on the campus’ implementation of safety measures. Participants recommended limiting in-person classes and lectures and ensuring that students have options for attending online classes, including flexible classes such as prerecorded classes and a hybrid of Zoom live and prerecorded classes. In cases of in-person classes, participants recommended the university limit interactions to small groups, place markers on the ground 6 feet apart, and put up Plexiglas to serve as protectors.

#### COVID-19 Education and Training

To increase access to COVID19 safety information on campus, participants recommended workshops and training to ensure that the campus community is on the same page despite sociocultural and political differences. Training would help decrease fears or concerns related to COVID-19. A staff member suggested, “teaching people [that] sometimes as soon as you sneeze [it could be] allergies, and not to directly assume its COVID.” Participants recommended that the campus community develop and implement mandatory modules on COVID-19 safety measures and provide information on how to support vulnerable populations on campus (e.g., how to deal with mask-related anxiety or claustrophobia). Campaigns on face covering and social distancing and providing credit or extension courses could increase widespread adoption of safety measures.

#### Flexible Classrooms and Office Spaces

Participants also discussed the need to “redesign the entire campus,” including classroom and offices. The university was perceived as a “city in and of itself,” with limited space, especially in classrooms, presenting challenges for accommodating social distancing measures. Participants noted that flexibility is needed to adapt class delivery and employees’ work. They discussed a hybrid classroom, alternating students for in-person instruction, and holding all large lectures online. Staff and faculty recommended eliminating micromanagement and expectations that people work on site, and “trust employees to be working [at home].” Employees who are able to work from home have had to “mitigate COVID and Zoom fatigue.”

## Discussion

This research provides insight into perceptions of safety behaviors and recommendations for their dissemination in reducing COVID-19 in IHEs. Public research universities, known for their ability to generate and transmit new knowledge through research and education, are well positioned to address the community health needs in the current pandemic. The community of scholars and critical thinkers within these institutions can play a significant role in unpacking the norms, values, and meaning systems shaping attitudes around COVID-19 safety measures and their use.

An important finding of our study is the role of sociocultural factors in COVID-19 perceptions and behaviors. Values of personal freedom and social consciousness shape decisions to engage (or not) in public health safety measures. Social and cultural factors (i.e., norms and values) hold both collective and individual meaning and ultimately influence individual-level decisions reducing or increasing virus spread. The actions of some, specifically those who hold personal freedom as their organizing principle, ultimately jeopardize the health of all. According to McLeroy et al. (1988), “it may not be necessary to ethically justify restriction of individual freedoms when the exercise of those freedoms imposes a clear harm to others” (p. 369).

As participants in our study recommended, a combination of top-down and ground-up solutions focused on community building and school spirit might be an effective strategy for adoption of COVID-19 safety measures. For example, top-down approaches can include campus administration developing guidelines, implementing them, and enforcing campus-wide use of face coverings, as well as investing in masks and their distribution in an effort to increase access to face coverings on campus. Grassroot approaches can include bringing students, staff, and faculty together to identify ways to use mask wearing and face coverings to build community and school spirit.

Another way to address differing values in the context of the current pandemic is for IHEs to lead the way in modeling behavior. Public research institutions should partner with public health departments, city and county officials, and health-care systems to educate the public on COVID-19. The approach can be one of unification bringing communities together around a shared purpose and vision.

Across the United States and around the globe, universities and public health systems are working together to frame messaging around collective efforts with the purpose of reducing virus spread and promoting health equity (National Institutes of Health, 2020). However, in the context of the United States and its current political climate (Green et al., 2020), the messaging needs to find common ground between those prioritizing personal liberty and those prioritizing social responsibility (Chung et al., 2020). CDC messaging, based on scientific evidence rather than personal/collective beliefs, may not be effective for both types of thinking.

Additionally, public health messaging fails to capture individualized experiences with safety measures. As participants noted, some people might have different physical and/or psychological experiences when wearing a mask (e.g., feelings of claustrophobia). What universities can do is partner with departments of public health to rethink messaging to incorporate individual experiences and promote alternative COVID-19 safety measures.

Furthermore, IHEs need to integrate environmental and health courses into existing curricula, making courses relevant to students’ real-life experiences, strengthening environmental responses to infectious diseases (e.g., hand washing, disinfectant use), and incorporating online mental health and medical services into existing student support services (Toquero, 2020). Our findings encourage IHEs to think beyond students’ needs: Faculty and staff also need access to environmental and health education, which could be done through training modules and extension courses.

We would ultimately like to emphasize the importance of IHEs increasing access to mental health-care services for the entire campus community, including students, staff, and faculty. The pandemic has been stressful for all members of the campus community—many students have transitioned to living and studying at home with family, and staff and faculty must often work and care for family, assist with children’s educational needs, and simultaneously attend meetings and meet deadlines during business hours. Acknowledging and addressing such stress is critical to flatten the emotional distress curve (Kaslow et al., 2020) and mental illness (Pfefferbaum & North, 2020) linked to the pandemic.

### Limitations

As with all qualitative studies, the findings are not generalizable and should be interpreted within the context of the following limitations. Conducting qualitative research in a defined community risks anonymity and may increase socially desirable responses. To reduce this possibility, we recruited potential participants through numerous social networks as well as strategized team members’ involvement in data collection. A second limitation was the over-recruitment of students into the focus groups. Unlike previous efforts, nearly all students recruited into the study attended a focus group; student focus groups were quite large. A third limitation is that the research was conducted prior to the emergency approval of the COVID-19 vaccines. In summer 2020, it was unclear when vaccines would be approved. Thus, we did not engage in discussions about returning to campus in the context of widespread access to COVID-19 vaccines.

### Conclusion

As campuses reopen, IHEs need to attend to collective needs and promote adequate and timely use of COVID-19 public health measures (Vindrola-Padros et al., 2020). This article advocates bringing together campus and public health leadership with grassroots leadership efforts to develop a shared vision and incorporate values that speak to all within the campus and surrounding communities.
